# Dexamethasone restores blood–brain barrier integrity in an *in vitro* heatstroke model

**DOI:** 10.1371/journal.pone.0352334

**Published:** 2026-06-29

**Authors:** Kazuaki Okamura, Karoline Pichlerova, Takayuki Matsuo, Daisuke Watanabe, William D. Cutts, Brandon J. Kim, Yoichi Morofuji

**Affiliations:** 1 Department of Neurosurgery, Nagasaki University Graduate School of Biomedical Sciences, Nagasaki, Japan; 2 Institute of Neuroimmunology, Slovak Academy of Sciences, Bratislava, Slovakia; 3 Department of Medical Pharmacology, Nagasaki University Graduate School of Biomedical Sciences, Nagasaki, Japan; 4 BBB Laboratory, PharmaCo-Cell Co., Ltd., Nagasaki City, Japan; 5 Department of Biological Sciences, University of Texas at Dallas, Richardson, Texas, United States of America; 6 Department of Neurosurgery, Showa Medical University School of Medicine, Tokyo, Japan; Gifu University School of Medicine Graduate School of Medicine: Gifu Daigaku Igakubu Daigakuin Igakukei Kenkyuka, JAPAN

## Abstract

Heat-related diseases and their treatments are becoming the center of focus due to global warming resulting in rising global temperatures. Heatstroke is the most hazardous condition of heat-related diseases, which when left untreated leads to death. One of the main characteristics of heatstroke is the dysfunction of the central nervous system. In this study, we established an *in vitro* heatstroke model of the blood–brain barrier (BBB) consisting of endothelial cells and pericytes. Following heat exposure at 43°C for 3 h, the model failed to recover during the subsequent 24 h regeneration period. The damage was shown by decreased transendothelial resistance (p < 0,0001) and confirmed by permeability assays and immunohistochemistry with *in silico* analysis. We subsequently evaluated the effect of dexamethasone in our heatstroke model. Administration of dexamethasone post-heatstroke alleviated BBB damage during the regeneration period, by increasing transendothelial electrical resistance and ZO-1 expression while reducing BBB permeability. Our findings suggest that dexamethasone reduces heatstroke damage at the BBB in *in vitro* conditions.

## Introduction

Global warming and the resulting increasing temperatures are a global health concern, due to its role in heat-related illnesses. Heatwaves were responsible for around 150 thousand deaths globally, per heat season, between the years 1990 and 2019 [1]. Hyperthermia is a common clinical feature of heat-related illnesses. Heatstroke is a life-threatening condition and the last stage in the spectrum of heat-related illnesses. Clinically, heatstroke is characterized by extreme hyperthermia with a core body temperature exceeding 40°C, central nervous system (CNS) dysfunction, and a history of exposure to hot and humid environments or excessive physical exertion [2]. Extensive heat-induced tissue injury, coagulopathies, and an immunity response (i.e., cytokines release) are leading to the development of a systemic inflammatory response syndrome, resulting into long-term multi-organ damage [3]. Heatstroke is generally classified into environmental and exertional heatstroke according to its cause. In case of environmental heatstroke are elderly and prepubertal children the most vulnerable populations. The course of heatstroke damage and the resulting death is similar for environmental and exertional heatstroke. Heatstroke damage to the central nervous system, is also facilitated by the negative impact on the different interfaces of the brain, such as the blood brain-barrier (BBB).

**Fig 1:**
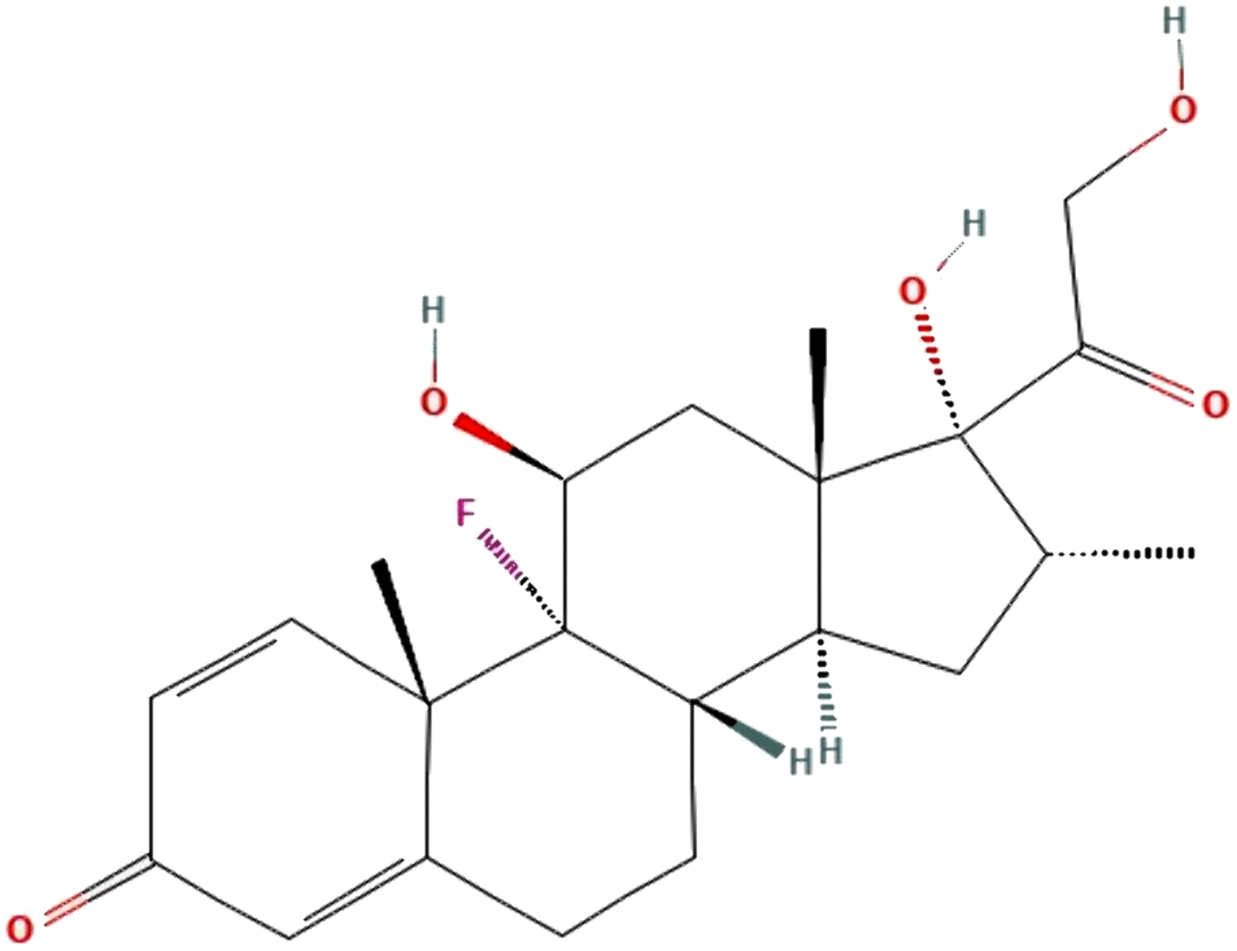
Chemical structure of dexamethasone. Illustrated chemical structure of dexamethasone (C_22_H_29_FO_5_), with depicted bonds (carbon in black, fluorine in purple, hydrogen in grey and oxygen in red).

Dexamethasone (DEX; Fig 1) is a synthetic glucocorticoid, used in a wide range of clinical conditions mainly, due to its anti-inflammatory and immunosuppressive effects. DEX exerts its effects through glucocorticoid receptor-mediated signaling pathways. DEX regulates expression of genes directly, or indirectly via transcription factors, of glucocorticoid response genes [4]. In addition, DEX activates non-genomic signaling pathways through glucocorticoid receptors, including kinase activation and endothelial nitric oxide synthase signaling, resulting in altered blood flow and reduced vascular inflammation [5]. DEX is currently being used to treat various conditions ranging from mild disorders (i.e., eczema) to severe diseases (i.e., glioblastoma) [6]. As a broad anti-inflammatory agent, DEX has been investigated in various neurological and systemic disorders, either as monotherapy or in combination with other treatments, with mixed clinical outcomes [7–9]. Previous *in vitro* and *in vivo* studies have demonstrated that DEX improves BBB barrier properties and reduces BBB permeability under both physiological and pathological conditions, including inflammation and tumor-associated BBB dysfunction [10,11]. However, the effects of DEX on BBB dysfunction under heatstroke conditions remain unclear, as previous studies have primarily focused on *in vivo* models [12].

In the present study, we established a rat *in vitro* heatstroke model of the BBB containing endothelial cells and pericytes. The effects of DEX at the site of BBB in the heatstroke model was subsequently evaluated. The effects of DEX on BBB dysfunction in the heatstroke model were subsequently evaluated. Our findings demonstrate that post-heatstroke administration of DEX restores BBB barrier properties.

## Materials and methods

### Materials

The reagents bought from Sigma (St. Louis, MO, USA) were Dulbecco's Modified Eagle's Medium/ Nutrient Mixture F-12 Ham (DMEM F-12; D0697), Evans’ Blue (E2129), Fluorescein sodium salt (F6377), Dulbecco’s PBS (D8537), Calcium Chloride (499609), Magnesium Chloride (M8266), glucose (G8270), HEPES (H3375), Paraformaldehyde (158127), Triton-X 100 (T8787), Bovine Serum Albumin (A7030). The Vectashield antifade mounting medium with DAPI (H-1200–10) was obtained from Vector laboratories (Newark, CA, USA). ZO-1 monoclonal primary antibodies (ZO1-1A12) and Alexa Fluor 488-conjugated donkey anti-mouse antibodies (A21202) were bought from Invitrogen (Waltham, MA, USA). Dexamethasone (S1322) was obtained from Selleck (Houston, TX, USA). The *in vitro* models (RBT-24H) were purchased from PharmaCo-Cell (PharmaCo-Cell Co., Ltd., Nagasaki, Japan).

### Preparation of the *in vitro* BBB model

The *in vitro* BBB model was prepared according to the method described by Nakagawa et al. [13]. Briefly, primary rat brain capillary endothelial cells (RBECs), pericytes, and astrocytes were isolated from Wistar rats and cultured under standard conditions.

RBECs were cultured in DMEM/F12 supplemented with 10% plasma-derived serum, basic fibroblast growth factor (1.5 ng/ml), insulin (5 µg/ml), transferrin (5 µg/ml), heparin (100 µg/ml), sodium selenite (5 ng/ml), gentamycin (50 µg/ml), and puromycin (4 µg/ml) at 37°C in 5% CO_2_/95% air. Pericytes were maintained in DMEM supplemented with 10% fetal bovine serum and antibiotics. Astrocytes were isolated from neonatal Wistar rat cortices and cultured in DMEM containing 10% fetal bovine serum.

Cells were seeded at the following densities: endothelial cells, 1.5 × 10^5^ cells/cm2; pericytes and astrocytes, 1.5 × 10^4^ cells/cm2.

For the experiments, a commercially available *in vitro* BBB model consisting of rat endothelial cells and pericytes was obtained from PharmaCo-Cell (PharmaCo-Cell Co., Ltd., Nagasaki, Japan) and prepared according to the manufacturer’s protocol. Prior to experiments, inserts containing endothelial cells and pericytes were transferred into wells without astrocytes and maintained at 37°C in 5% CO_2_ for 5 days. Medium exchange was performed one day before experiments to remove hydrocortisone from the culture system.

### Evaluation of the barrier integrity

To evaluate the barrier integrity, transendothelial electrical resistance (TEER) was measured by an EVOM resistance meter and Endohm chamber (World Precision Instruments, Sarasota, FL, USA). The background resistance value (blank insert) was subtracted from values of inserts with cells. TEER value [Ω*cm^2^] is calculated as TEER after the removal of the background, times size of insert (0,33 cm^2^). In selected experiments, TEER was continuously monitored every 30 min using a cellZscope system (nanoAnalytics GmbH, Münster, Germany). TEER data are presented as percentages relative to baseline values.

### Construction of the heatstroke *in vitro BBB* model

One day prior to experiments, was the culture medium changed to remove hydrocortisone from the BBB models. Baseline TEER values were measured using an Endohm chamber before heat exposure.

To characterize heat stress-induced BBB dysfunction, experiments were performed using three types of BBB models: endothelial cell monoculture (E00), endothelial cell–pericyte coculture (EP0), and endothelial cell–pericyte–astrocyte coculture (EPA). Samples were divided into controls (37°C) and heat load (41–43°C) groups.

Preliminary experiments were conducted under various heat load conditions to determine the optimal parameters for irreversible BBB injury. Heat exposure durations ranged from 1 to 24 h, followed by recovery periods of up to 70 h at 37°C in 5% CO_2_. Experimental replicates of different models ranged from 3 to 6 per condition.

At 41°C, E00 models were exposed to heat load for 1, 3, or 6 h followed by 24 h recovery. At 42°C, E00, EP0, and EPA models were exposed to heat load for 4 h (EPA), 6 h (E00, EP0, EPA), 24 h (EPA), with recovery periods ranging from 24 h (E00, EP0, EPA) to 70 h (E00, EP0). At 43°C, EP0 models were exposed to heat load for 3–4 h followed with a 24 h recovery period.

### Heatstroke induction and DEX treatment

TEER was measured before the experiments and samples were divided into groups (n = 5). DEX, in the concentration of 100 nM, was administered to both luminal and abluminal sites of the model, after the heatstroke was carried out. Heatstroke conditions were induced by exposing the *in vitro* BBB models to 43°C in 5% CO_2_ for 3 h.

### Transendothelial permeability

Following the experiments, permeability of Evans’ blue-albumin (EBA, MW: 67 kDa) and sodium fluorescein (Na-F, MW: 376 Da) across the endothelial monolayer was determined and used as an index of transcellular and paracellular transport [14,15]. Cell culture inserts were transferred to 24-well plates containing 0.9 mL assay buffer (Dulbecco’s PBS (D-PBS) containing 0.9 mM CaCl_2_, 0.5 mM MgCl_2_, 4.5 g/L glucose, and 10 mM HEPES; pH 7.4). The luminal culture medium was replaced with 0.2 mL of assay buffer containing 10 μg/mL Na-F and 165 μg/mL Evans’ blue-albumin (MW 67 kDa). Fifteen minutes after Na-F and Evans´ blue addition, were the inserts transferred to new wells containing the assay buffer. Na-F concentration in the collected samples was measured using a multiwell spectrophotometer (Tecan Spark, Zürich, Switzerland; excitation wavelength: 485 nm) with the emission measurement at 535 nm. The absorbance of Evans´ blue was at 595 nm. P_app_ (cm/s), the apparent permeability coefficient, was derived from Fick´s Law and calculated as described in Youdim et al. [16].

### Immunostaining

For the post-experimental observation of the tight junction marker, were cells stained with monoclonal primary antibodies against ZO-1 (Invitrogen, Waltham, MA, USA) with a dilution of 1:100. Secondary antibodies were Alexa Fluor 488-conjugated donkey anti-mouse (Invitrogen, Waltham, MA, USA) with a dilution of 1:1000. After the experiments were the pericytes on the membrane removed with a Q-tip, and the endothelial cells in inserts washed once with phosphate-buffered saline (PBS). The endothelial cells were subsequently fixed in 3% paraformaldehyde in PBS for 10 min at room temperature. After fixation were cells washed three times with PBS and permeabilized with 0.1% Triton-X 100 for 10 min at room temperature. Cells were washed with PBS three times and blocked with 3% bovine serum albumin in PBS for one hour at room temperature. The cells were washed three times with PBS and incubated with primary anti-ZO-1 antibodies in blocking buffer overnight at 4 °C. Subsequently, were cells washed three times with PBS and incubated with secondary antibodies in PBS, for 1 h at room temperature. After a three-time wash with PBS were the membranes cut out of the inserts and mounted with the Vectashield antifade mounting medium with DAPI (Vector laboratories, Newark, CA, USA). The staining was examined under an EVOS® FL Cell Imaging System (Thermo Fisher Scientific, Eugene, USA).

### Statistical analysis

All data presented are expressed as means ± S.E.M. Values were compared using 2-way ANOVA statistical tests. In cases where 2way ANOVA was ruled out, the Šídák's multiple comparisons test was carried out instead. Changes were considered statistically significant at p < 0.0001.

### *In silico* analysis of ZO-1 tight junction marker

The JAnaP (RRID: SCR_026027) script is publicly accessible and available for download at https://github.com/StrokaLab/JAnaP. The program was used as described in Gray et al. [17]. For the JAnaP analysis 21–54 cells per image (amount of waypointed cells depends on amount of cells and integrity) were manually defined via waypointing. The pictures were processed by two different persons, to remove user bias. A threshold value was determined from a representative cell from the control condition (37°C) and then used to filter out background fluorescence in each image. The JAnaP “Fast Class” feature was utilized to analyze all waypointed images. The main parameter of the measurement was the signal continuity of the marker.

## Results

### Construction of an *in vitro* heatstroke model

**Fig 2:**
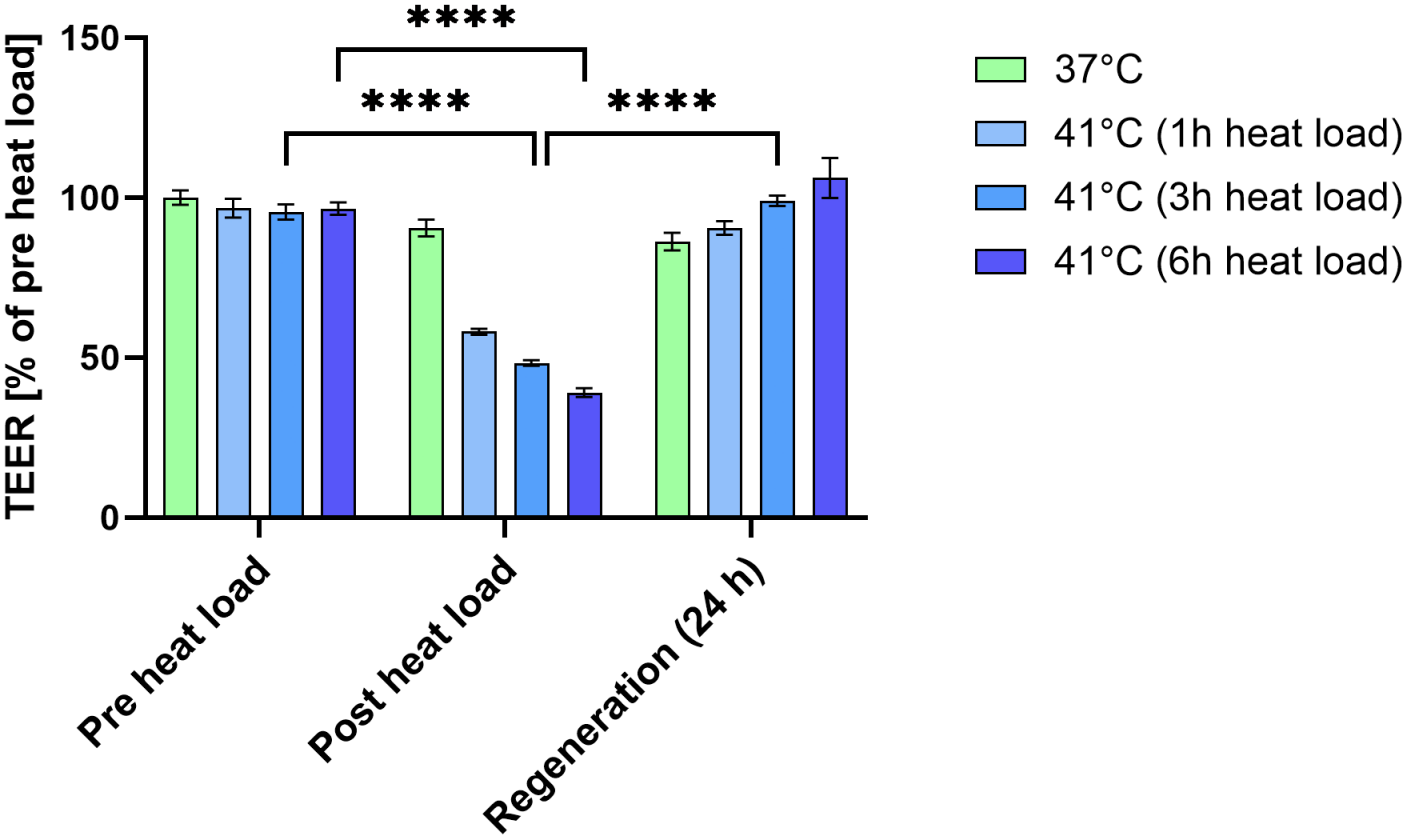
Influence of different times (1 h, 3 h, 6 h) of 41°C heat load, on the TEER of the E00 model. The decrease of TEER for different heat load times ranged between ~40-60%, with a subsequent recovery of the models after 24 h regeneration time, with an increase above the pre-heat load levels (~105%) in case of 6 h heat load. n = 5; 2-way ANOVA statistical test. **** p < 0.0001.

After the preparation of the *in vitro* model and removal of hydrocortisone, were cells incubated under different heat load conditions and subsequently regeneration was carried out under 37°C, 5% CO_2_ conditions. E00 models were exposed to 41°C for 1 h, 3 h and 6 h of heat load (n = 5). The subsequent regeneration time of the model was 24 h. TEER decrease ranged from 42% to 60% and was statistically significant (p < 0.0001) for 3 h and 6 h heat load conditions. After 24 h of regeneration at 37°C, TEER recovered to pre-heat load levels and in case of 6 h heat load was the TEER increased above pre-heat load levels (Fig 2).

**Fig 3:**
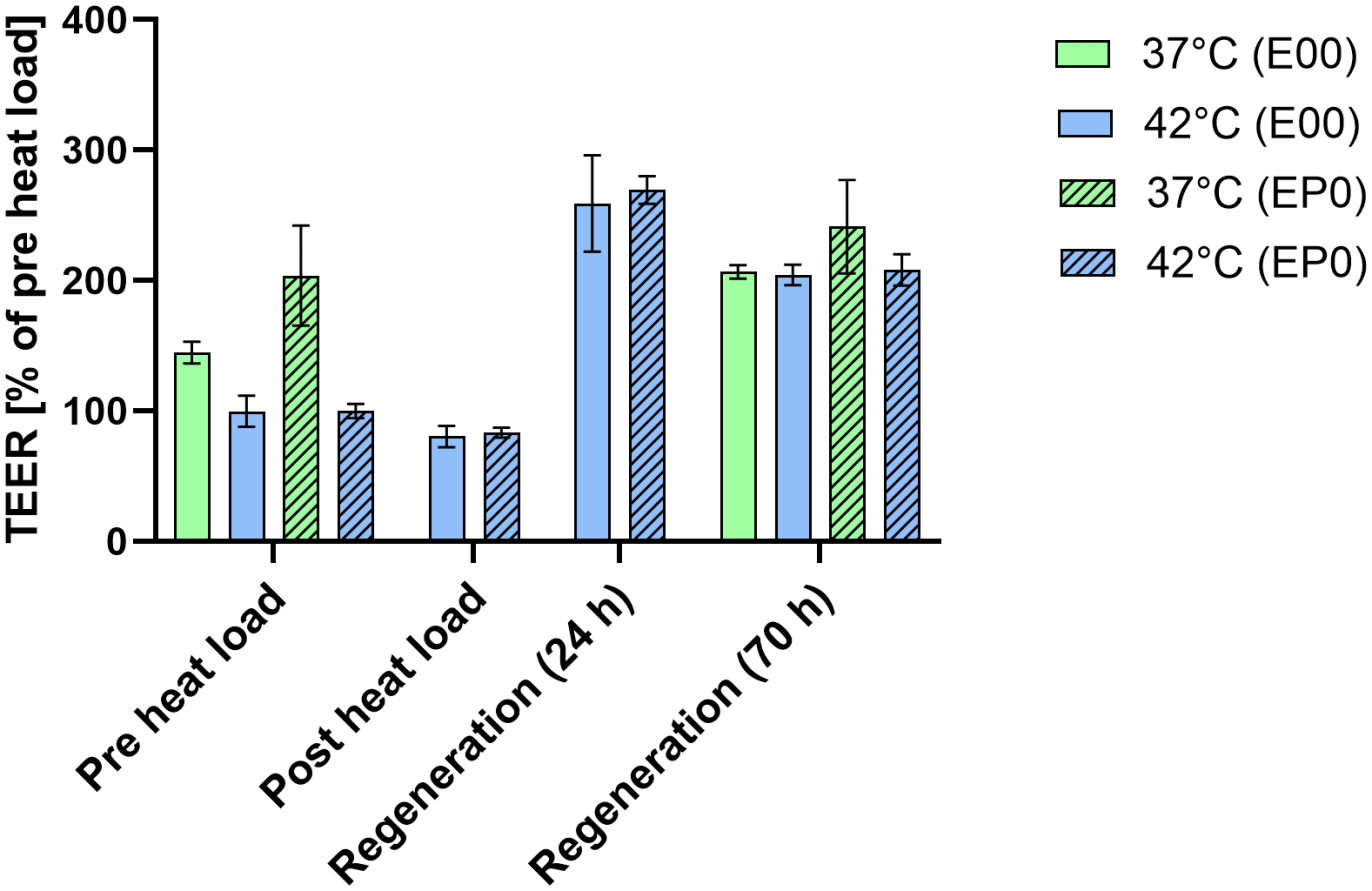
TEER decrease of E00, EP0 model after a 6 h heat load of 42°C. With a regeneration time of up to 70 h. Controls (37°C) were measured only at experiment start and end. TEER of heat load samples was measured every 30 minutes, with post heat load and 24 h, 70 h regeneration time demonstarted. TEER decreased slightly, with recovery to higher TEER levels than pre-heat load, with a slight decrease in TEER at the 70 h regeneration mark. n = 3; 2-way ANOVA statistical test. No significance.

At 42°C were the E00 and EP0 models exposed to a 6 h heat load with a subsequent 70 h recovery time (n = 3). In both models was the decrease of TEER after the heat load about 20% after 6 h, with models displaying robust recovery capabilities at 24 h and 70 h of regeneration. The models recovered post heat load to TEER values with an 250% increase, compared to pre-heat load values, at the 24 h recovery mark and remained elevated at 200% of pre-heat load values at the 70 h recovery mark (Fig 3).

**Fig 4:**
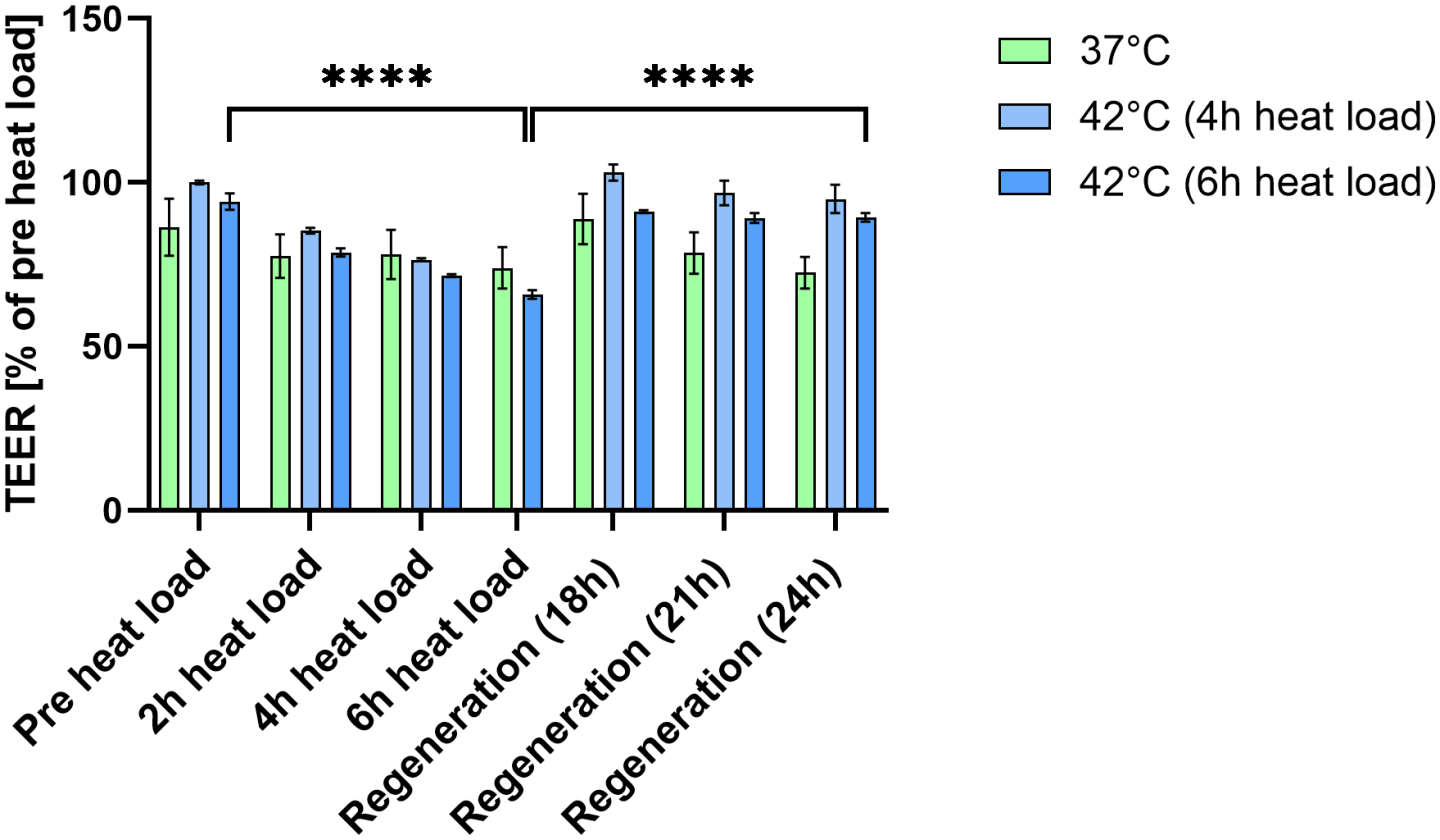
The effects on TEER of a 4 h and 6 h heat load of 42°C on the EPA model. After the TEER decrease, recovery was measured up to 24 h with increased TEER compared to pre-heat load levels. TEER decreased by approximately 25–30% after heat exposure and recovered to near baseline levels following 24 h regeneration. n = 3; 2-way ANOVA statistical test. **** p < 0.0001.

The EPA model was tested at 42°C for 4 h and 6 h (n = 3). For the 4 and 6 h heat load did TEER decrease by 25–30% immediately after heat load. The models were capable to recover to near pre-heat load levels (94% of pre-heat load levels for the 4 h heat load and 89% for the 6 h heat load) following the 24 h regeneration (Fig 4). Additionally, a test with 24 h, 42°C heat load n the EPA model was carried out (n = 3). The 24 h heat load resulted in a 55% TEER decrease of the model, with an impaired recovery after 24 h regeneration time. After recovery TEER remained at ~45% below pre-heat load levels (S1 Fig).

**Fig 5:**
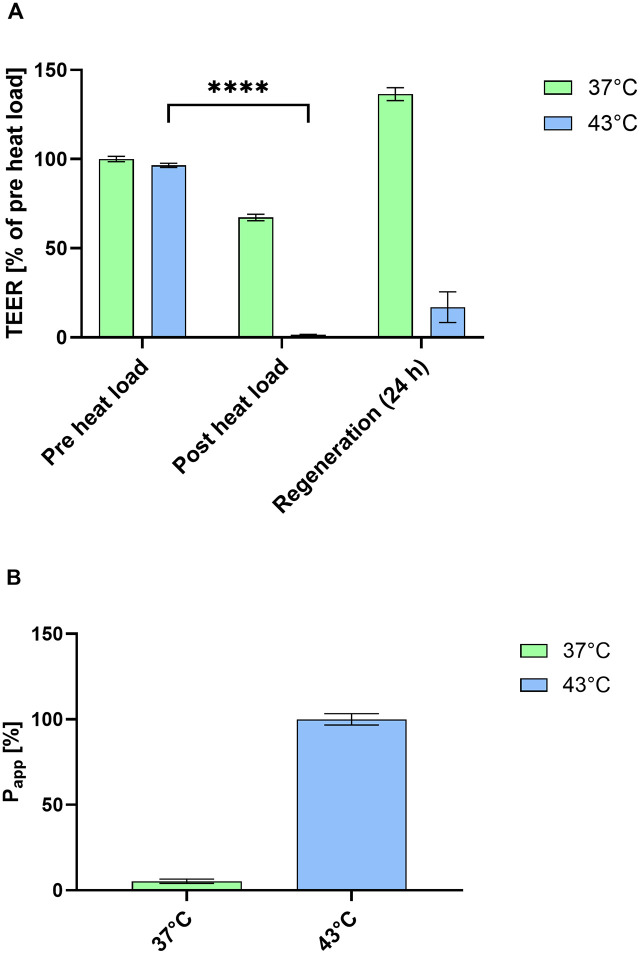
The effects of a 43°C, 4 h heat load on TEER and NaF permeability in the EP0 model. **A**) TEER measurements of the EP0 model comparing pre- and post-heat load TEER, with a significant decrease of TEER post heat load. The capabilities of regeneration were exhausted without a proper regeneration after 24 h, with only a slight TEER increase. n = 6; n = 3 for regeneration **B)** NaF permeability assay of control and heat loaded groups. The permeability increased substantially in comparison to controls. n = 3; Šidák's multiple comparisons test as statisical test (TEER measurements). **** p < 0.0001.

A durability test of the EPA model to an exposure of a 43°C heat load within a 6 h period was carried out (n = 4). The TEER decreased by 35% after at the 6 h time point. The capabilities to recover were not investigated in this test (S2 Fig). Similarly, was the EP0 model exposed to 43°C heat load conditions for the duration of 4 h (n = 6), with a regeneration duration of 24 h. TEER decreased significantly by 99% compared to pre-heat load levels (Fig 5). The permeability was assesed on half of the samples post heat load, with a substantial increase in NaF permeability (Fig 5). Regeneration was carried out on the second half of samples, with a 84% TEER decrease, compared to pre-heat load levels, after 24 h of regeneration (Fig 5).

The optimal conditions for the *in vitro* EP0 heatstroke model, were set to 3 hours of heat load at 43°C, after which the cells were not capable of regeneration (37°C for 24 h post heat load). The heatstroke damage to the cells and the regeneration incapability of the model were confirmed through its low TEER, high permeability with NaF and EBA assays, and tight junction marker decrease (ZO-1) post heatstroke and post regeneration.

### Heatstroke damage is alleviated with DEX in an *in vitro* model of the BBB

**Fig 6:**
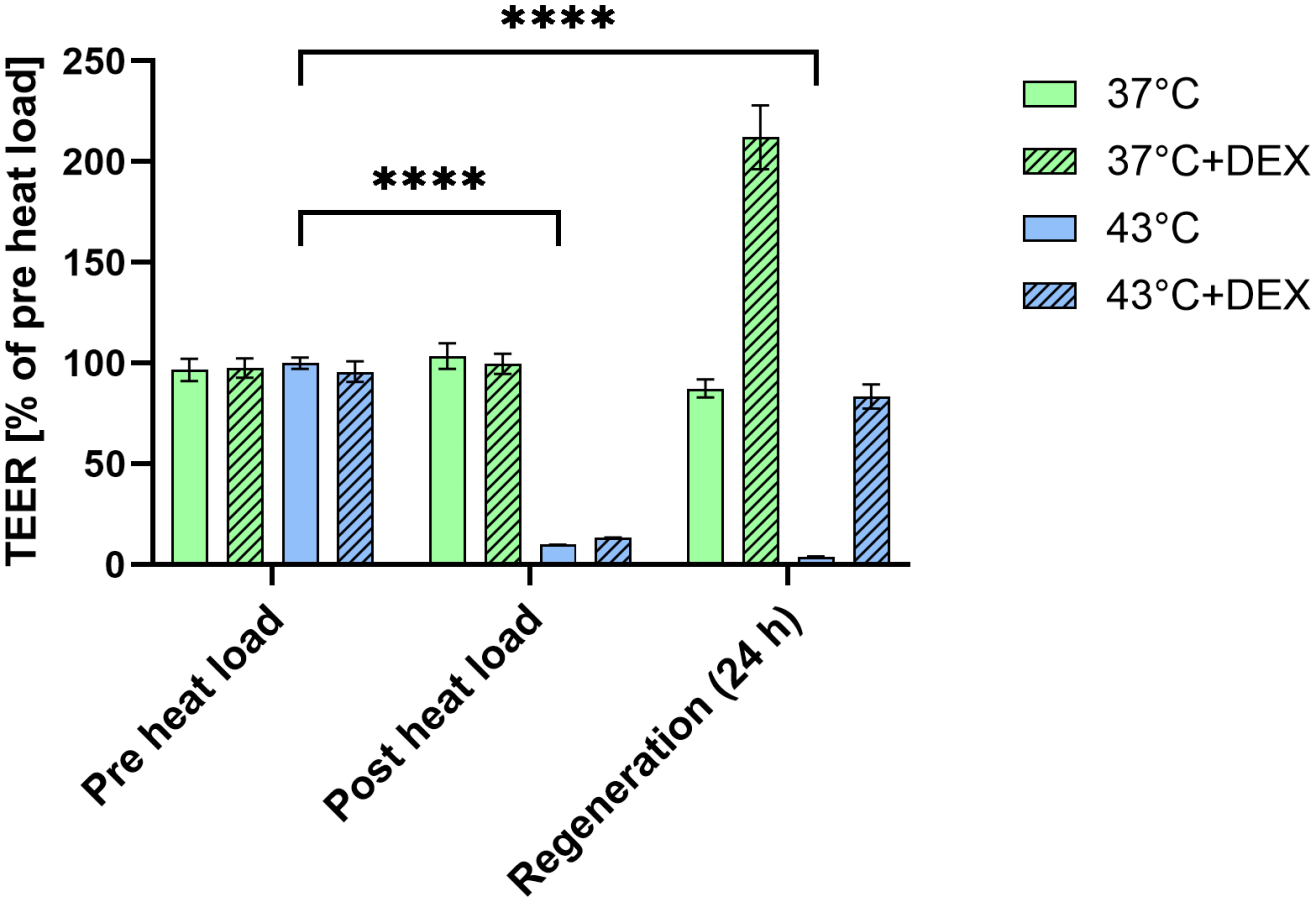
Comparison of TEER for the heatstroke model and the DEX treated samples. TEER measurements of the heatstroke model comparing pre- and post-heat load TEER and the models capabilities of regeneration (treated vs non-treated groups). Decrease of TEER was significant post-heat load with no capacity for recovery after 24 h of regeneration. The addition of DEX lead to an increase of TEER to pre-heat load levels, after 24 h regeneration. n = 5; 2-way ANOVA statistical test. **** p < 0.0001.

**Fig 7:**
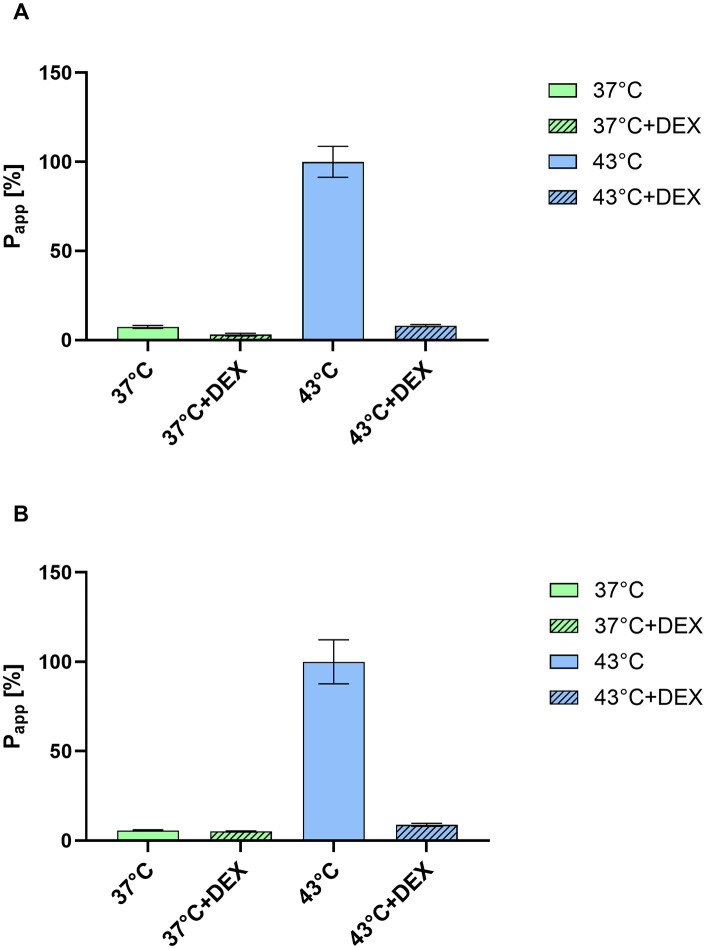
Comparison of the barrier permeability markers for the heatstroke model and the DEX treated samples. Permeability was measured post-regeneration. **A)** NaF permeability assay of control and heatstroke (treated vs non-treated) groups. **B)** EBA permeability assay of control and heatstroke (treated vs non-treated) groups. The decrease of barrier tightness and increase in permeability of the heatstroke model, after the regeneration time of 24 h, was substantial. Samples with added DEX reversed the leakiness of the barrier, having permeability coefficient similar to controls. n = 5.

**Fig 8:**
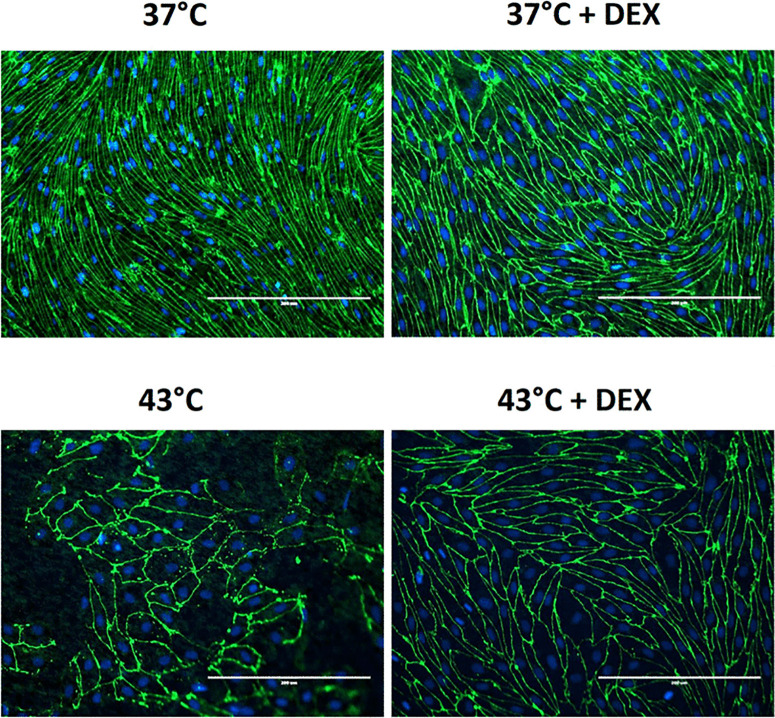
ZO-1 immunostaining of tight junctions of the controls (non-treated vs treated) and samples post-heat load (non-treated vs treated). ZO-1 protein is stained green and cell nuclei are stained blue. The scale in the pictures is 200 µm.

**Fig 9:**
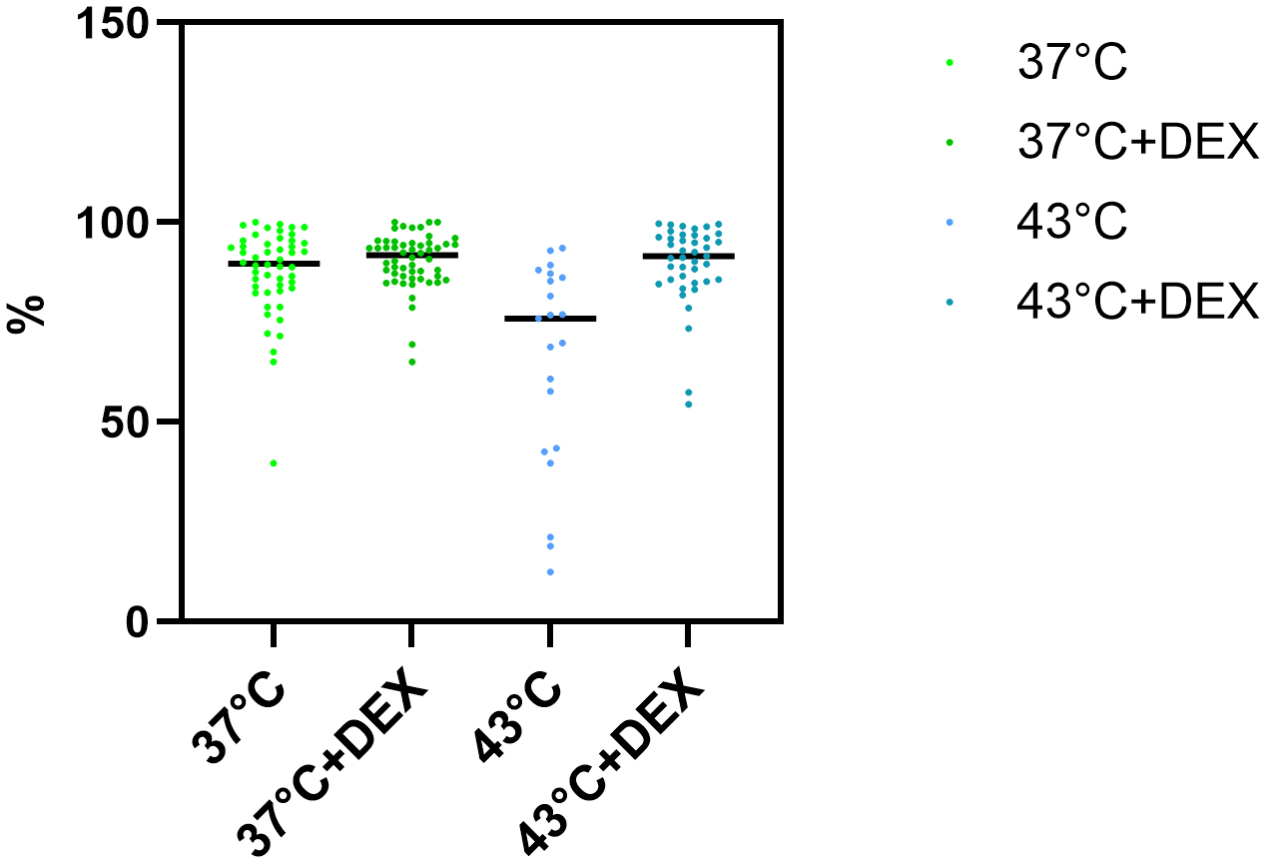
JAnaP analysis of the ZO-1 continuity from Fig 8 of all groups (controls and samples, with or without DEX). The continuity of ZO-1 is decreased in samples post-heat load, compared to controls. The ZO-1 continuity of with DEX treated post-heat load samples have a continuity similar to controls treated with DEX.

At the start were the heatstroke model samples divided into groups (n = 5), with an evenly distributed average starting TEER for each group. The experimental groups consisted of two control groups at 37°C (- DEX and + DEX groups) and two heatstroke groups. Similarly to control groups, was the first heatstroke group treated with dexamethasone post heatstroke, while the second group did not get DEX administered. TEER was measured post heatstroke, before the start of regeneration for all groups. Both heatstroke groups had significantly reduced TEER, post heat load, in comparison to control groups (Fig 6) (p < 0.0001). The integrity of the barrier (TEER, NaF, EBA assays, tight junction immunostaining) was measured for every group post regeneration. Control group (37°C) treated with DEX has shown increase in TEER, post regeneration, but the amount of ZO-1 tight junction markers decreased. Post heatstroke after 24 h regeneration, was TEER in the heatstroke group without DEX significantly reduced (p < 0.0001) (Fig 6), in comparison to both control groups. Similarly, were other barrier integrity factors (NaF, EPA and ZO-1) in the non-treated heatstroke group significantly reduced, in comparison to the control group (Figs 7 and 8). In case of the DEX treated heatstroke group, was TEER (Fig 6) and other barrier integrity markers (NaF and EBA) recovered to similar levels as non-DEX treated control (Fig 7) (p < 0.0001). In case of DEX treated heatstroke group were tight junctions damaged in a lesser extent than in the non-treated heatstroke group (Fig 8). The *in silico* method of JAnaP mapping was conducted to quantify the tight junctions in Fig 8. The continuity of the signal in the JAnaP analysis is a marker for the junction architecture disruption. In the JAnaP analysis is the continuity of the control group treated with DEX similar to control. The heatstroke group has a decreased average continuity, while the heatstroke group treated with DEX is having a similar continuity to the control (Fig 9).

## Discussion

The aim of the study was to establish an *in vitro* heatstroke model of the BBB, to study the effect of pharmacological agents on BBB integrity under heatstroke conditions. In the early phase several different types of heatstroke models were to define the conditions. The conditions tested in the model included the type of the model, time of heat load and the temperature. The sufficient heat load, leading to heatstroke in the *in vitro* model was determined by the incapability of the model to self-regenerate to pre-heat load levels and decreased barrier properties of the model (low TEER, high permeability). Post heat load recovery time was set to 24 h in cell growth physiological conditions (37°C, 5% CO_2_).

Currently, no proper BBB model of heatstroke, which would enable effective, cheap testing of drugs for their neuroprotective effects, prior to proceedings of *in vivo* tests, exists. In our *in vitro* heatstroke model did the temperatures range from 41°C to 43°C for the heat load and the time of the heat load from 1 h to 6 h, with focus on most-likely scenarios of clinical heatstroke in patients. The aim of the heatstroke model was to incorporate cellular components of the neurovascular unit (endothelial cells, pericytes, astrocytes), as hyperthermia is damaging endothelial cells and pericytes, which can results into damage and increased leakage of the BBB, while also influencing the behavior of astrocytes [18]. Astrocytes can play a dual role in heatstroke, while pericytes play an important role in stabilizing and ensuring the integrity of the BBB [19].

The first tests were set to 41°C heat load conditions on the E00 model. Even though the decrease of TEER was 60% in case of the heat load duration of 6 h, was the model was capable to sufficiently recover and exceed pre-heat load levels without any intervention (Fig 2). Due to the recovery capabilities of the model at 41°C heat load was the temperature of the heat load increased to 42°C. The heat load conditions of 42°C were tested on E00, EP0 and EPA models, with an average decreases of TEER between 15–30% for all models at 6 h heat load duration. In all cases were the models capable of subsequent recovery, surpassing pre-heat load TEER levels (Figs 3 and 4). In case of 70 h regeneration (Fig 3), the TEER decreased slightly at the 70 h mark, compared to 24 h regeneration, which can be a result from the models natural declines in TEER at later days of the cultivation of the model (according to the manufacturer is the time window for consisten experiments between day 5 and 7 of the cultivation). Heat load time was increased to 24 h on the EPA model, to test the durability capabilities of the model. After 24 h of 42°C heat load, did the TEER decrease by 55% in comparison to pre-heat load levels and the recovery of the model was impaired from the prolonged heat load. The TEER was not able to recover to pre-heat load levels, after regeneration, and had a decrease of 45% compared to pre-heat levels, but without further decline (S1 Fig). Since 41°C and 42°C models led to reversible heat damage at the BBB, with extensive heat load durations required to exhaust the recovery capabilities of the models (E00, EP0, EPA), a 43°C heat load model was investigated. The EPA model was tested at 43°C heat load, during the period of 6 h. TEER decreased by 35% after the 6 h duration, with the TEER levels having a similar decrease as the 42°C EPA model. As the decrease of TEER was not significant enough and the model behaved similar to 42°C heat load a recovery test was not carried out (S2 Fig). The higher durability and lower decrease in TEER of the EPA model in short heat load durations, and its subsequent incapability of recovery after a significant TEER decrease is supporting claims of astrocytes playing a possible role in protection and inflammation during hyperthermia [18]. Due to the higher durability of the EPA model, a durability test on the EP0 model in the 43°C heat load conditions, for a span of 3 h and 4 h, was carried out. The TEER of the model was decreased by 99% at the 4 h heat load duration and by 83% in case of the 3 h heat load period, with a significant increase of NaF permeability (for both durations) and EBA permeability (for 3 h duration). The regeneration capabilities of the models were heavily impaired, post regeneration, where the TEER of the model was decreased by 84% (in case of 4 h duration) and 94% (in case of 3 h duration), compared to pre-heat load levels (Figs 5 and 6). The higher recovery after 4 h versus 3 h at 43°C in the EP0 model likely reflects inter‑experiment biological variability and technical factors (e.g., culture age, replicate differences), rather than a true biological difference, as parallels, except one had a decrease post regeneration time.

Our EP0 heatstroke model was experimentally set up to 43°C, with the heat load time being 3 h and a 24 h post-heat load regeneration period, to more closely mimic clinically relevant cases of early heatstroke before the onset of significant inflammation, as longer periods of heat load in clinical situations, lead to multiorgan failure and death, instead of patient survival.

This condition was selected because it represented a consistent heat exposure that consistently induced severe and persistent BBB dysfunction, while maintaining structural integrity for subsequent therapeutic evaluation. Due to preliminary data from 41–42°C heat load models, showing models preserving recovery capabilities at short heat load duration, were shorter time points in 43°C EP0 model not investigated, and 3 h heat load was set as the shortest period in the heatstroke model. Studies are reporting diagnosed hyperthermia in the range of 40–43.6°C in patients with heatstroke [20,21], with rare cases experiencing hyperthermia up to 46,5°C [22]. The duration of untreated exertional or environmental heatstroke, ranges from minutes to hours, before the onset of multi organ failure, inflammation and eventually death from untreated heatstroke. The treatment of heatstroke in clinical cases ranges from several days to several weeks [23,24]. A study shows neuronal tissue changes in patients with deadly outcomes of extreme hyperthermia [25]. High hyperthermia at 43°C facilitates BBB breakdown that manifests as vasogenic cerebral edema [20] leading to a raised intracranial pressure and acute neurologic deterioration (seizures, coma) [25]. Neuroinflammation is also present in clinical patients with heatstroke, with neuroinflammation having an onset of several hours post heatstroke. The disruption of the BBB correlates with worse outcomes in clinical studies [26]. Our *in vitro* BBB model mirrors the disruption of the BBB in an early onset heatstroke (3 h), without inflammation.

DEX as an inflammatory drug, might lead to a slower onset of neuroinflammation and may play a neuroprotective role at the BBB. In clinical practice do the non-genomic effects of DEX have a rapid onset of up to 2 h (depending on the route of admission) [27]. Peak plasma concentrations occur around 1 h after oral dosing, with symptomatic improvement in cerebral edema patients being within hours, with the highest benefit being around the 24 h – 48 h mark [28]. The effect of DEX *in in vitro* conditions is, similar to other drugs, instant and the concentration of DEX in *in vitro* conditions is also constant, as the break down of drugs in *in vitro* conditions is significantly lower.

Studies focusing on the effect of DEX, specifically on the BBB, in heatstroke conditions have not been carried out. In our experiments were the effects of DEX, on the BBB, assessed in physiological and heat-stress conditions. The chosen concentration of 100 nM DEX reflects the low-dose treatments tested for heatstroke in animals [29] and is also aligning with DEX's limited brain penetration, since P-glycoprotein (P-gp/ABCB1) at the BBB actively effluxes DEX [30]. Additionally, is the 100 nM concentration of DEX commonly used in other experimental settings and in which a higher concentrations of DEX can be cytotoxic [31,32]. The concentration curve of dexamethasone and its cytotoxicity on our specific model was not studied. For a clinically relevant scenario was DEX administered early post heat load, assessing its BBB protective capabilities in the subsequent regeneration. In case of physiological conditions did DEX further increase TEER and the barrier integrity (Figs 6 and 7), while the increase in ZO-1 tight junction protein was minimal (Fig 8). The increase in barrier integrity at 37°C correlates with other groups findings of DEX having a positive effect on the BBB in physiological conditions. Post heat load was DEX able to restore barrier integrity and restore the BBB properties to pre-stress levels in both TEER and ZO-1 tight junction markers (Fig 8).

JAnaP analysis was the choice in our experiments due to higher number of waypoints and lower time consumption, in comparison to IJOQ [33]. *In silico* analysis of ZO-1 tight junctions is showing a recovery of marker continuity to the same level as controls (Fig 9). Our results are supporting the claim of a possible neuroprotective role of DEX in heatstroke at the site of BBB, in case of early intervention.

The main focus of the study was the acute phase of heatstroke and related to this the clinicaly relevant first 24 h of treatment post heatstroke. The recovery was assessed up to 24 h, with additional later time points such as 48 h and 72 h not included. Therefore, we cannot directly define the full regeneration or recovery time course beyond 24 h. However, the observed positive effect of DEX at 24 h suggests an early beneficial response.

### Limits of this study

Several limitations of this study should be acknowledged. The heatstroke model is missing one component of the neurovascular unit (astrocytes), due to the prolonged period of heat load needed, to significantly impact the barrier properties in the model. The severity, an recovery capabilities, of shorter heat load durations (< 3 h) in the 43°C, EP0 model were not studied. The study focused on one concentration of DEX and a dose–dependent analysis, respectively cytotoxic concentrations of DEX were not characterized in the model. Additionally, was the main focus of the an early intervention and the crucial first 24 h of recovery post-heatstroke, which did not account for heavy neuroinflammation, and delayed medical interventions. The prolonged recovery times of 48 h and 72 h are missing from our study, as an prolonged cultivation of the model leads to a natural decline in barrier properties, resulting in on extended regeneration time. The heatstroke model and study was carried out in *in vitro* conditions, which did not account for factors such as hemodynamics, neuroinflammation and immune response. The model should serve as a suggestion for possible animal and clinical studies, before introduction of the therapy to clinical practice.

## Conclusion

An *in vitro* heatstroke model of the blood-brain barrier, containing rat endothelial cells and rat pericytes was established. The effects of the synthetic glucocorticoid dexamethasone, as an off-target, were investigated in the *in vitro* heatstroke model of the BBB. The heat damage to the BBB (decreased TEER, loss of tight-junction ZO-1 protein) was mitigated by administration of dexamethasone immediately after heat exposure. The attenuation of heat damage, at the BBB, by dexamethasone might lead to a better outcome.

## Supporting information

S1 Fig The effects of a 42°C, 24 h heat load on the EPA model and its subsequent regeneration. The prolonged heat load halved TEER of the EPA model. The self-recovery capabilities of the model were depleted post-heat load, as it was not capable to recover TEER to pre-heat load levels after 24 h regeneration. n = 3.
(TIF)

S2 Fig The effects of a 43°C, 6 h heat load on the EPA model. TEER was halved at increased heat load of 43°C, during a 6 h period. The model struggled in self-recovery as TEER stagnated during the regeneration duration.n = 4.
(TIF)
