# Supplementary material for: Dexamethasone restores blood–brain barrier integrity in an in vitro heatstroke model
Source: PLoS One. 2026 Jun 29;21(6):e0352334. doi: 10.1371/journal.pone.0352334 (PMC13313372; doi:10.1371/journal.pone.0352334)
Supplement: Attachment — Submitted filename: Response to Reviewers YM.docx [file pone.0352334.s004.docx]

Dear Reviewers,

We thank you for your consideration of our manuscript and for the constructive comments. Below are our responses to each comment and descriptions of the changes made in the revised manuscript.

**Reviewer 1**

(1) The authors mention preliminary experiments that led to the chosen conditions. It would be helpful to describe these preliminary experiments within the context of model developments. Are the effects of elevated temperature time-dependent? At what stage do they become toxic to the cells? A cell viability assay with different incubation times at 43 C of less than 3 h would be useful.

Response: We added important preliminary experiments (41–43°C) to “Results” and “Supporting Information” that compare E00, EP0, and EPA models, with different heat loads. Heat load of 43°C was carried out on EPA and EP0 models, with different durations. The thought process behind the choice of heatstroke model is disscused in the “Discussion” section. Results show that the heat load effect is dependent on the type of the model, including cell types of the BBB (the EPA model being most durable). 42°C caused cytotoxicity with prolonged exposure (24h) in EPA, while exposure time of 6h did not have cytotoxic effect on E00, EP0 models; 43°C produced less damage in EPA (6h duration), but was cytotoxic to EP0 at shorter durations (3h, 4h). We deprioritized 1–2 h heat‑load durations, due our models showing more significant BBB damage at 3–4 h heat load in other preliminary experiments. This decision was taking into account the unreliable 43°C EPA results, with no recovery duration. We are acknowledging the missing test in the study and the point was added to the “Limitations of this study” section.

(2) Different recovery times would also be clinically relevant. Please show the time-dependent effects of recovery times of 24h, 48h, 72 h.

Response: We focused in our study mainly on the crucial first 24 h of the critical care post-heatstroke; no full 48 h or 72 h recovery experiments were performed. We now explicitly state this limitation in “Limitations of this study” section. Additionally, TEER is stable between the days 5 and 7 of experiments, according to the manufacturer, which can lead to unreliable TEER measurements, due to the natural TEER decrease in the model in longer periods of regeneration.

(3) Only one concentration of dexamethasone was used in the experiments. The effects of dexamethasone are known to be dose-dependent. This could also be demonstrated in viability tests or barrier property measurements.

Response: A full dexamethasone dose–response or cytotoxicity curve was not carried out in our study. We clarified in the “Discussion” section, that the concentration of 100 nM DEX was chosen based on literature and BBB penetration considerations, and we acknowledged the lack of dose–dependence data on our specific model in the section “Limitations of this study”, with a consideration for future studies.

(4) Abstract: please describe the methods used and the results in more detail.

Response: We added TEER measurement, permeability assays and immunohistochemistry with an *in silico* analysis as methods used to demonstrate damage to the BBB in the “Abstract” section. Results, in the “Abstract” section, were described in more detail – capability of dexamethasone to reverse the damage at the BBB post-heatstroke, in all studied factors (TEER, permeability, increased tight junction marker expression).

(5) Methods: Please provide the catalog numbers of all reagents and cells used. “*In vitro* BBB model” this section requires more detail. Although the *in vitro* model is commercially available, a precise step-by-step description of the cell culture would still be important.

Response: We have updated the “Materials” subsection to include producer names and catalog numbers for all reagents, antibodies, and cell lines used in the experiments. The “Preparation of the *In vitro* BBB model” subsection in the “Material and methods” section now provides a brief protocol with seeding densities, with the step-by-step protocol being cited in study in the subsection.

**Reviewer 2**

Thank you for the endorsement; we appreciate the positive evaluation and have addressed the specific suggestions below.

(1) Second “blood-brain barrier” in Abstract is preferable to be “BBB”.

Response: We changed the second “blood-brain barrier” in Abstract to “BBB”, and made the use of abbreviations (BBB, DEX) more consistent in the study.

(2) It is preferable to show the structure of dexamethasone.

Response: We added a description of the chemical structure of dexamethasone as Figure 1 in the “Introduction” section of the paper.

(3) The establishment of an *in vitro* heatstroke model was assessed based on its low TEER, high permeability as measured by NaF and EBA assays, and the decrease in the tight junction marker ZO-1 after heatstroke and after regeneration. However, although the model is evaluated as “heatstroke,” does it truly recapitulate actual heatstroke conditions?

Response: We acknowledge the limitations of *in vitro* heatstroke models, in fully reproducing clinical and physiological responses to heatstroke, such as inflammation and hemodynamic changes. We have added a paragraph in the “Discussion” section, where we are discussing features of clinical heatstroke including recorded core temperatures (40-43.6°C; in extreme cases up to 45°C), duration (several hours), irreversible BBB damage (usually caused by onset of inflammation) and secondary neuroinflammation. In our model we focused on hallmarks of BBB injury occurring during heatstroke (decreased TEER, increased permeability, reduced tight junctions). The model focuses on early heatstroke, before the onset of inflammation. The drawbacks of the model (lack of inflammation, hemodynamic changes) were mentioned in “Limitations of this study”.

(4) Does dexamethasone exert its effects rapidly?

Response: We added a paragraph in the “Discussion” section briefly explaining this topic. In short, the effects of dexamethasone in an *in vitro* environment are immediate with a defined concentration. In case of *in viv*o experiments and clinical practice, the onset of dexamethasone effect depends on the doses, route of administration and permeability into the brain. The onset in the clinical environment is about 1 h - 2 h with the highest efficacy appearing at the 24 - 48 h mark post administration. Half-life and breakdown of dexamethasone depends on the hepatic function of the patient.

The manuscript-wide changes are as follows:

(1) Figures and legends updated to incorporate new panels (Figs. 1–5, Supplementary Figs. 1-2) and statistical details (tests used, n values, p-values).
(2) Added preliminary experiments, their description (in “Materials and methods”, “Results” section) and the thought process behind the creation of the heatstroke model (in “Discussion” section).
(3) Methods and materials expanded with reagent catalog numbers and producer, equipment models, and a brief protocol on construction of the BBB model, with a citation with full point-to-point protocol for isolation of cells and production of the BBB model.

(4) Added points to “Discussion” section about the choice of conditions for the heatstroke model. Compared the BBB heatstroke model to clinical relevancy and added a paragraph of onset of DEX effects in *in vitro* and in clinical setups. Added a paragraph discussing the chosen 100 nM concentration in experiments, accounting for low dose treatments of heatstroke in animal studies, its limited brain penetration in clinical setup and DEX's cytotoxicity in high concentrations studied in literature. Added a paragraph discussing the early acute phase of heatstroke, which is related to the clinical relevant 24 h.

(5) Added a “Limitations of this study” section, describing the limits of our study (missing astrocytes in the model, missing dose-dependency and cytotoxic experiments of DEX, focus on early clinical intervention, not accounting for neuroinflammation and delayed medical interventions, being an *in vitro* experiment as a preliminary model for *in viv*o and clinical studies)

(6) Language edits throughout the publication for clarity.

Summary of major experimental additions

- Expanded methods and added catalog numbers; included chemical dexamethasone structure (Fig 1)
- Time-course of heat exposures between 41°C-43°C with heat load durations ranging from 3 h - 24 h and recovery period of 24 h - 70 h — Figs 2-4
- Increased duration (4 h) of 43°C heat load on EP0 model and subsequent 24 h regeneration (Fig 5)
- Increased 42°C heat load (period of 24 h) on EPA model and its regeneration with a duration of 24 h (S1 Fig)
- Test of 43°C heat load on the EPA model over the course of 6 h, with no recovery duration (S2 Fig)

We believe these additions directly address the reviewers’ concerns by (1) clarifying how experimental conditions of the heatstroke model were chosen (2) providing insight into chosen conditions mimicking clinical cases and (3) providing the requested methodological details. The dose–response and full onset-kinetics of dexamethasone were not performed and are noted as limitations. We welcome any further suggestions.

Sincerely,

Yoichi Morofuji

**Yoichi Morofuji, M.D., Ph.D.**

Professor and Chair

Department of Neurosurgery
Showa Medical University School of Medicine
1-5-8 Hatanodai, Shinagawa-ku, Tokyo 142-8666 Japan
Tel: +81-3-3784-8605
Fax: +81-3-3784-8432

Email: [morofujiyoichi@gmail.com](mailto:morofujiyoichi@gmail.com); [youichi.morofuji@showa-u.ac.jp](mailto:youichi.morofuji@med.showa-u.ac.jp)
