# Supplementary material for: Dexamethasone restores blood–brain barrier integrity in an in vitro heatstroke model
Source: PLoS One. 2026 Jun 29;21(6):e0352334. doi: 10.1371/journal.pone.0352334 (PMC13313372; doi:10.1371/journal.pone.0352334)
Supplement: Peer review history

# Peer review: Decision Letter 0

**DOI:** 10.1371/journal.pone.0352334.r001

**Authors:** Hideshi Okada

24 Apr 2026

PONE-D-26-13479

Dexamethasone Restores Blood–Brain Barrier Integrity in an In Vitro Heatstroke Model

PLOS One

Dear Dr. Morofuji,

Thank you for submitting your manuscript to PLOS ONE. After careful consideration, we feel that it has merit but does not fully meet PLOS ONE’s publication criteria as it currently stands. Therefore, we invite you to submit a revised version of the manuscript that addresses the points raised during the review process.

This manuscript addresses an important and timely topic, namely the role of blood–brain barrier dysfunction in heatstroke and the potential protective effects of dexamethasone. The establishment of an in vitro BBB heatstroke model is of interest and may provide a useful platform for mechanistic investigation.

However, both reviewers have raised substantial concerns regarding the current level of experimental validation and methodological detail. I concur with their assessment that, while the study is promising, the current version is not yet sufficient for publication without substantial revision and additional clarification.

In particular, the major issues relate to:

1. insufficient characterization of the in vitro heatstroke model
2. limited exploration of time- and dose-dependent effects
3. lack of methodological transparency and reproducibility

Please address in accordance carefully with reviewers` comments in a revised version.

In summary, while this study addresses an important question and presents potentially valuable findings, substantial revision is required to strengthen the experimental foundation and improve clarity and reproducibility.

We therefore invite you to submit a revised version addressing all comments raised by the reviewers and the editor.

Please submit your revised manuscript by Jun 08 2026 11:59PM. If you will need more time than this to complete your revisions, please reply to this message or contact the journal office at plosone@plos.org. When you're ready to submit your revision, log on to https://www.editorialmanager.com/pone/ and select the 'Submissions Needing Revision' folder to locate your manuscript file.

Please include the following items when submitting your revised manuscript:

- A letter that responds to each point raised by the academic editor and reviewer(s). You should upload this letter as a separate file labeled 'Response to Reviewers'.
- A marked-up copy of your manuscript that highlights changes made to the original version. You should upload this as a separate file labeled 'Revised Manuscript with Track Changes'.
- An unmarked version of your revised paper without tracked changes. You should upload this as a separate file labeled 'Manuscript'.

If you would like to make changes to your financial disclosure, please include your updated statement in your cover letter. Guidelines for resubmitting your figure files are available below the reviewer comments at the end of this letter.

If applicable, we recommend that you deposit your laboratory protocols in protocols.io to enhance the reproducibility of your results. Protocols.io assigns your protocol its own identifier (DOI) so that it can be cited independently in the future. For instructions see: https://journals.plos.org/plosone/s/submission-guidelines#loc-laboratory-protocols. Additionally, PLOS ONE offers an option for publishing peer-reviewed Lab Protocol articles, which describe protocols hosted on protocols.io. Read more information on sharing protocols at https://plos.org/protocols?utm_medium=editorial-email&utm_source=authorletters&utm_campaign=protocols.

As the corresponding author, your ORCID iD is verified in the submission system and will appear in the published article. PLOS supports the use of ORCID, and we encourage all coauthors to register for an ORCID iD and use it as well. Please encourage your coauthors to verify their ORCID iD within the submission system before final acceptance, as unverified ORCID iDs will not appear in the published article. *Only*  the individual author can complete the verification step; PLOS staff *cannot*  verify ORCID iDs on behalf of authors.

We look forward to receiving your revised manuscript.

Kind regards,

Hideshi Okada, M.D., Ph.D.

Academic Editor

PLOS One

Journal Requirements:

When submitting your revision, we need you to address these additional requirements.

1. Please ensure that your manuscript meets PLOS ONE's style requirements, including those for file naming. The PLOS ONE style templates can be found at

https://journals.plos.org/plosone/s/file?id=wjVg/PLOSOne_formatting_sample_main_body.pdf and

https://journals.plos.org/plosone/s/file?id=ba62/PLOSOne_formatting_sample_title_authors_affiliations.pdf

2.We note that you have included the phrase “data not shown” in your manuscript. Unfortunately, this does not meet our data sharing requirements. PLOS does not permit references to inaccessible data. We require that authors provide all relevant data within the paper, Supporting Information files, or in an acceptable, public repository. Please add a citation to support this phrase or upload the data that corresponds with these findings to a stable repository (such as Figshare or Dryad) and provide and URLs, DOIs, or accession numbers that may be used to access these data. Or, if the data are not a core part of the research being presented in your study, we ask that you remove the phrase that refers to these data.

3. Please note that PLOS One has specific guidelines on code sharing for submissions in which author-generated code underpins the findings in the manuscript. In these cases, we expect all author-generated code to be made available without restrictions upon publication of the work. Please review our guidelines at https://journals.plos.org/plosone/s/materials-and-software-sharing#loc-sharing-code and ensure that your code is shared in a way that follows best practice and facilitates reproducibility and reuse.

4. We note that your Data Availability Statement is currently as follows: "All relevant data are within the paper."

Please confirm at this time whether or not your submission contains all raw data required to replicate the results of your study. Authors must share the “minimal data set” for their submission. PLOS defines the minimal data set to consist of the data required to replicate all study findings reported in the article, as well as related metadata and methods (https://journals.plos.org/plosone/s/data-availability#loc-minimal-data-set-definition).

For example, authors should submit the following data:

- The values behind the means, standard deviations and other measures reported;

- The values used to build graphs;

- The points extracted from images for analysis.

Authors do not need to submit their entire data set if only a portion of the data was used in the reported study.

If your submission does not contain these data, please either upload them as Supporting Information files or deposit them to a stable, public repository and provide us with the relevant URLs, DOIs, or accession numbers. For a list of recommended repositories, please see https://journals.plos.org/plosone/s/recommended-repositories.

If there are ethical or legal restrictions on sharing a de-identified data set, please explain them in detail (e.g., data contain potentially sensitive information, data are owned by a third-party organization, etc.) and who has imposed them (e.g., an ethics committee). Please also provide contact information for a data access committee, ethics committee, or other institutional body to which data requests may be sent. If data are owned by a third party, please indicate how others may request data access.

5. If the reviewer comments include a recommendation to cite specific previously published works, please review and evaluate these publications to determine whether they are relevant and should be cited. There is no requirement to cite these works unless the editor has indicated otherwise.

Additional Editor Comments:

Thank you for submitting your manuscript entitled “Dexamethasone Restores Blood–Brain Barrier Integrity in an In Vitro Heatstroke Model” to PLOS ONE.

This manuscript addresses an important and timely topic, namely the role of blood–brain barrier dysfunction in heatstroke and the potential protective effects of dexamethasone. The establishment of an in vitro BBB heatstroke model is of interest and may provide a useful platform for mechanistic investigation.

However, both reviewers have raised substantial concerns regarding the current level of experimental validation and methodological detail. I concur with their assessment that, while the study is promising, the current version is not yet sufficient for publication without substantial revision and clarification.

In particular, the major issues relate to:

1. insufficient characterization of the in vitro heatstroke model

2. limited exploration of time- and dose-dependent effects

3. lack of methodological transparency and reproducibility

Please revise the manuscript carefully in accordance with the reviewers’ comments.

In summary, while this study addresses an important question and presents potentially valuable findings, substantial revision is required to strengthen the experimental foundation and improve clarity and reproducibility. We therefore invite you to submit a revised version addressing all comments raised by the reviewers and the editor.

We look forward to receiving your revised manuscript.

[Note: HTML markup is below. Please do not edit.]

Reviewers' comments:

Reviewer's Responses to Questions

**Comments to the Author**

1. Is the manuscript technically sound, and do the data support the conclusions?

The manuscript must describe a technically sound piece of scientific research with data that supports the conclusions. Experiments must have been conducted rigorously, with appropriate controls, replication, and sample sizes. The conclusions must be drawn appropriately based on the data presented.

Reviewer #1: Partly

Reviewer #2: Yes

**********

2. Has the statistical analysis been performed appropriately and rigorously?

Reviewer #1: Yes

Reviewer #2: Yes

**********

3. Have the authors made all data underlying the findings in their manuscript fully available?

The PLOS Data policy requires authors to make all data underlying the findings described in their manuscript fully available without restriction, with rare exception (please refer to the Data Availability Statement in the manuscript PDF file). The data should be provided as part of the manuscript or its supporting information, or deposited to a public repository. For example, in addition to summary statistics, the data points behind means, medians and variance measures should be available. If there are restrictions on publicly sharing data—e.g. participant privacy or use of data from a third party—those must be specified.

Reviewer #1: No

Reviewer #2: Yes

**********

4. Is the manuscript presented in an intelligible fashion and written in standard English?

PLOS ONE does not copyedit accepted manuscripts, so the language in submitted articles must be clear, correct, and unambiguous. Any typographical or grammatical errors should be corrected at revision, so please note any specific errors here.

Reviewer #1: Yes

Reviewer #2: Yes

**********

5. Review Comments to the Author

Please use the space provided to explain your answers to the questions above. You may also include additional comments for the author, including concerns about dual publication, research ethics, or publication ethics. (Please upload your review as an attachment if it exceeds 20,000 characters)

Reviewer #1: The manuscript by Okamura et al. with a title “Dexamethasone Restores Blood-Brain Barrier Integrity in an In Vitro Heatstroke Model” describes the protective effects of dexamethasone treatment in heatstroke using a rat blood-brain barrier model based on brain microvascular endothelial cells co-cultured with pericytes. The topic of this study is of interest because in vivo studies have shown that dexamethasone has protective effects in heatstroke. In vitro investigation of this topic allows for a more detailed elucidation of the underlying molecular mechanisms. The authors demonstrate the effects on transendothelial electrical resistance (TEER) and permeability of Evans blue albumin and sodium fluorescein, as well as on ZO-1 staining of tight junctions. The authors used a 3 h-heatstroke at 43 C as the heatstroke model with or without dexamethasone treatment for 24 h. The results show significant changes and a protective effect of dexamethasone. The authors point out, however, that this is the first in vitro model for heatstroke and present only limited results. More detailed studies are needed for a comprehensive initial characterization of the model.

1. The authors mention preliminary experiments that led to the chosen conditions. It would be helpful to describe these preliminary experiments within the context of model developments. Are the effects of elevated temperature time-dependent? At what stage do they become toxic to the cells? A cell viability assay with different incubation times at 43 C of less than 3 h would be useful.

2. Different recovery times would also be clinically relevant. Please show the time-dependent effects of recovery times of 24h, 48h, 72 h.

3. Only one concentration of dexamethasone was used in the experiments. The effects of dexamethasone are known to be dose-dependent. This could also be demonstrated in viability tests or barrier property measurements.

4. Abstract: please describe the methods used and the results in more detail.

5. Methods: Please provide the catalog numbers of all reagents and cells used. “In vitro BBB model” this section requires more detail. Although the in vitro model is commercially available, a precise step-by-step description of the cell culture would still be important.

Reviewer #2: The manuscript described the effects of dexamethasone at the site of BBB in the heatstroke. The authors showed the establishment of heatstroke model and the result of dexamethasone. Thus, these findings will be useful for the treatment of the dysfunction of the central nervous system due to heatstroke. Therefore, the manuscript is not too excellent to be published. In other words, the manuscript is so excellent that it should be published.

Comments

(1) Second “blood-brain barrier” in Abstract is preferable to be “BBB”.

(2) It is preferable to show the structure of dexamethasone.

(3) The establishment of an in vitro heatstroke model was assessed based on its low TEER, high permeability as measured by NaF and EBA assays, and the decrease in the tight junction marker ZO-1 after heatstroke and after regeneration. However, although the model is evaluated as “heatstroke,” does it truly recapitulate actual heatstroke conditions?

(4) Does dexamethasone exert its effects rapidly?

That is all.

**********

6. PLOS authors have the option to publish the peer review history of their article (what does this mean?). If published, this will include your full peer review and any attached files.

If you choose “no”, your identity will remain anonymous but your review may still be made public.

**Do you want your identity to be public for this peer review?**  For information about this choice, including consent withdrawal, please see our Privacy Policy.

Reviewer #1: No

Reviewer #2: No

**********

[NOTE: If reviewer comments were submitted as an attachment file, they will be attached to this email and accessible via the submission site. Please log into your account, locate the manuscript record, and check for the action link "View Attachments". If this link does not appear, there are no attachment files.]

To ensure your figures meet our technical requirements, please review our figure guidelines: https://journals.plos.org/plosone/s/figures

You may also use PLOS’s free figure tool, NAAS, to help you prepare publication quality figures: https://journals.plos.org/plosone/s/figures#loc-tools-for-figure-preparation.

NAAS will assess whether your figures meet our technical requirements by comparing each figure against our figure specifications.

# Author comment: Author response to Decision Letter 1

**DOI:** 10.1371/journal.pone.0352334.r002

2 Jun 2026

We thank the Editor and Reviewers for their valuable comments. Detailed point-by-point responses are provided in the attached response letter.

Attachment Submitted filename: Response to Reviewers YM.docx

# Peer review: Decision Letter 1

**DOI:** 10.1371/journal.pone.0352334.r003

**Authors:** Hideshi Okada, Hideshi Okada

9 Jun 2026

Dexamethasone Restores Blood–Brain Barrier Integrity in an In Vitro Heatstroke Model

PONE-D-26-13479R1

Dear Dr. Morofuji,

We’re pleased to inform you that your manuscript has been judged scientifically suitable for publication and will be formally accepted for publication once it meets all outstanding technical requirements.

Within one week, you’ll receive an e-mail detailing the required amendments. When these have been addressed, you’ll receive a formal acceptance letter and your manuscript will be scheduled for publication.

An invoice will be generated when your article is formally accepted. Please note, if your institution has a publishing partnership with PLOS and your article meets the relevant criteria, all or part of your publication costs will be covered. Please make sure your user information is up-to-date by logging into Editorial Manager at Editorial Manager® and clicking the ‘Update My Information' link at the top of the page. For questions related to billing, please contact billing support.

If your institution or institutions have a press office, please notify them about your upcoming paper to help maximize its impact. If they’ll be preparing press materials, please inform our press team as soon as possible -- no later than 48 hours after receiving the formal acceptance. Your manuscript will remain under strict press embargo until 2 pm Eastern Time on the date of publication. For more information, please contact onepress@plos.org.

Kind regards,

Hideshi Okada, M.D., Ph.D.

Academic Editor

PLOS One

Additional Editor Comments (optional):

Both reviewers have now evaluated the revised manuscript and are satisfied with the authors' responses and revisions. I agree that the manuscript is scientifically sound and suitable for publication in PLOS ONE.

I am therefore pleased to inform you that your manuscript has been accepted for publication.

Reviewers' comments:

Reviewer's Responses to Questions

**Comments to the Author**

1. If the authors have adequately addressed your comments raised in a previous round of review and you feel that this manuscript is now acceptable for publication, you may indicate that here to bypass the “Comments to the Author” section, enter your conflict of interest statement in the “Confidential to Editor” section, and submit your "Accept" recommendation.

Reviewer #1: All comments have been addressed

Reviewer #2: All comments have been addressed

**********

2. Is the manuscript technically sound, and do the data support the conclusions?

The manuscript must describe a technically sound piece of scientific research with data that supports the conclusions. Experiments must have been conducted rigorously, with appropriate controls, replication, and sample sizes. The conclusions must be drawn appropriately based on the data presented.

Reviewer #1: Yes

Reviewer #2: Yes

**********

3. Has the statistical analysis been performed appropriately and rigorously?

Reviewer #1: Yes

Reviewer #2: Yes

**********

4. Have the authors made all data underlying the findings in their manuscript fully available?

The PLOS Data policy requires authors to make all data underlying the findings described in their manuscript fully available without restriction, with rare exception (please refer to the Data Availability Statement in the manuscript PDF file). The data should be provided as part of the manuscript or its supporting information, or deposited to a public repository. For example, in addition to summary statistics, the data points behind means, medians and variance measures should be available. If there are restrictions on publicly sharing data—e.g. participant privacy or use of data from a third party—those must be specified.

Reviewer #1: Yes

Reviewer #2: Yes

**********

5. Is the manuscript presented in an intelligible fashion and written in standard English?

PLOS ONE does not copyedit accepted manuscripts, so the language in submitted articles must be clear, correct, and unambiguous. Any typographical or grammatical errors should be corrected at revision, so please note any specific errors here.

Reviewer #1: Yes

Reviewer #2: Yes

**********

6. Review Comments to the Author

Please use the space provided to explain your answers to the questions above. You may also include additional comments for the author, including concerns about dual publication, research ethics, or publication ethics. (Please upload your review as an attachment if it exceeds 20,000 characters)

Reviewer #1: The authors have revised the manuscript in accordance with the reviewers’ comments. I have no further concerns.

Reviewer #2: The manuscript is well revised and the authors have adequately addressed all of the reviewer’s previous comments. The study presents a well-designed in vitro blood–brain barrier model of heatstroke and provides convincing evidence of BBB dysfunction under heat stress conditions, as well as the protective effects of dexamethasone. The results are clearly described, and the conclusions are supported by the experimental data. Overall, the manuscript is scientifically sound and suitable for publication in its present form. Please publish it in this form.

**********

7. PLOS authors have the option to publish the peer review history of their article (what does this mean?). If published, this will include your full peer review and any attached files.

If you choose “no”, your identity will remain anonymous but your review may still be made public.

**Do you want your identity to be public for this peer review?**  For information about this choice, including consent withdrawal, please see our Privacy Policy.

Reviewer #1: No

Reviewer #2: No

**********

# Editor report: Acceptance letter

**DOI:** 10.1371/journal.pone.0352334.r004

**Authors:** Hideshi Okada, Hideshi Okada

PONE-D-26-13479R1

PLOS One

Dear Dr. Morofuji,

I'm pleased to inform you that your manuscript has been deemed suitable for publication in PLOS One. Congratulations! Your manuscript is now being handed over to our production team.

At this stage, our production department will prepare your paper for publication. This includes ensuring the following:

* All references, tables, and figures are properly cited

* All relevant supporting information is included in the manuscript submission,

* There are no issues that prevent the paper from being properly typeset

You will receive further instructions from the production team, including instructions on how to review your proof when it is ready. Please keep in mind that we are working through a large volume of accepted articles, so please give us a few days to review your paper and let you know the next and final steps.

Lastly, if your institution or institutions have a press office, please let them know about your upcoming paper now to help maximize its impact. If they'll be preparing press materials, please inform our press team within the next 48 hours. Your manuscript will remain under strict press embargo until 2 pm Eastern Time on the date of publication. For more information, please contact onepress@plos.org.

You will receive an invoice from PLOS for your publication fee after your manuscript has reached the completed accept phase. If you receive an email requesting payment before acceptance or for any other service, this may be a phishing scheme. Learn how to identify phishing emails and protect your accounts at https://explore.plos.org/phishing.

If we can help with anything else, please email us at customercare@plos.org.

Thank you for submitting your work to PLOS ONE and supporting open access.

Kind regards,

PLOS ONE Editorial Office Staff

on behalf of

Dr. Hideshi Okada

Academic Editor

PLOS One
